# Metronomic Chemotherapy in Elderly Patients

**DOI:** 10.1007/s11912-024-01505-w

**Published:** 2024-03-07

**Authors:** Arianna Bandini, Pasquale Fabio Calabrò, Marta Banchi, Paola Orlandi, Guido Bocci

**Affiliations:** Department of Clinical and Experimental Medicine, University of Pisa, Via Roma 55, 56126 Pisa, Italy

**Keywords:** Metronomic chemotherapy, Elderly patients, Low-dose regimen, Geriatric oncology

## Abstract

**Purpose of Review:**

This review describes the most relevant studies found in the scientific literature regarding metronomic chemotherapy (MCT) in the geriatric oncology population to support its use as a feasible treatment of care in the frail elderly patients.

**Recent Findings:**

Recent years have seen a reevaluation of cancer chemotherapeutic drugs and MCT is an emerging schedule in phase II and III clinical trials.

**Summary:**

Ageing is one of the risk factors for the development of cancer, the incidence of whom increases dramatically in people who live longer. To date, standard oncological protocols involve chemotherapeutic drugs in short cycles of therapy at the maximum tolerated dose (MTD). Although these therapeutic regimens may be successful, they can cause important adverse drug reactions, especially in elderly or frail patients. MCT is a different modality of delivery of chemotherapeutic drugs (frequent low dose for prolonged time) and it looks at the overcoming of the limitations and disadvantages of MTD, in particular the toxicity aspect. We reviewed the experience of clinicians who have used MCT in clinical trials enrolling elderly patients with different cancer types.

## Introduction

Ageing is the main risk factor for cancer and for the complications of cytotoxic chemotherapy, and this is one of the reasons why the prognosis of many tumors worsens with age [1]. This paper explores the tolerance and the effectiveness of metronomic chemotherapy (MCT) in the older aged person.

### Metronomic Chemotherapy

There is no single definition of "elderly" person, but in general, the most commonly used age threshold for identifying elderly patients in subgroup analyses of clinical trials is 65 years [2]. The available data suggest that metronomic chemotherapy may be tolerable and effective also in the so called "frail patients" whose functional status prevents the use of chemotherapy in full doses. Although there is still no general agreement on the definition and assessment of frailty, the American Medical Association has described as "frail" the patients that present the most difficult and complicated management issues for clinicians and other medical professionals [3]. Frailty reflects a geriatric clinical condition characterized by severe vulnerability and inability to maintain homeostasis in the face of stressors. This is mainly due to the reduction in resilience due to limited physiological reserves and impaired organ function. [4, 5].

To date, clinical management regimens in oncology involve may chemotherapeutic drugs, alone or in combination with other agents, such as targeted therapies or immunotherapy. Systemic treatment may be used for locally advanced or metastatic malignancies. Most chemotherapeutic drugs are intended to be cytotoxic for fast-proliferating cells and they are often administered intermittently at the highest tolerable doses indicated with the term "maximum tolerated dose" (MTD). These therapeutic regimens succeed through the direct cytotoxicity of rapidly dividing tumor cells, but at the same time they cause serious adverse drug reactions, especially in elderly or frail patients [6, 7••].

In recent years there has been a re-evaluation of how chemotherapeutic drugs could be administered. “Metronomic Chemotherapy” (MCT) represents an emergent trend in cancer chemotherapy drug delivery. The term MCT is commonly used to describe the frequent and regular, even on a daily schedule (at one tenth to one-third of MTD), for extended time, without breaks [8••]. As it has been noted by Bocci and Kerbel, the definition of metronomic chemotherapy should include achievement of prolonged plasma concentration of active drug levels thanks to the prolonged administration of chemotherapy in low dose [8••]. The constant exposure of tumor cells to the drug has also an impact on the tumor microenvironment. This includes suppression of tumor angiogenesis and restoration of anti-tumor immune response [9, 10].

**Fig. 1:**
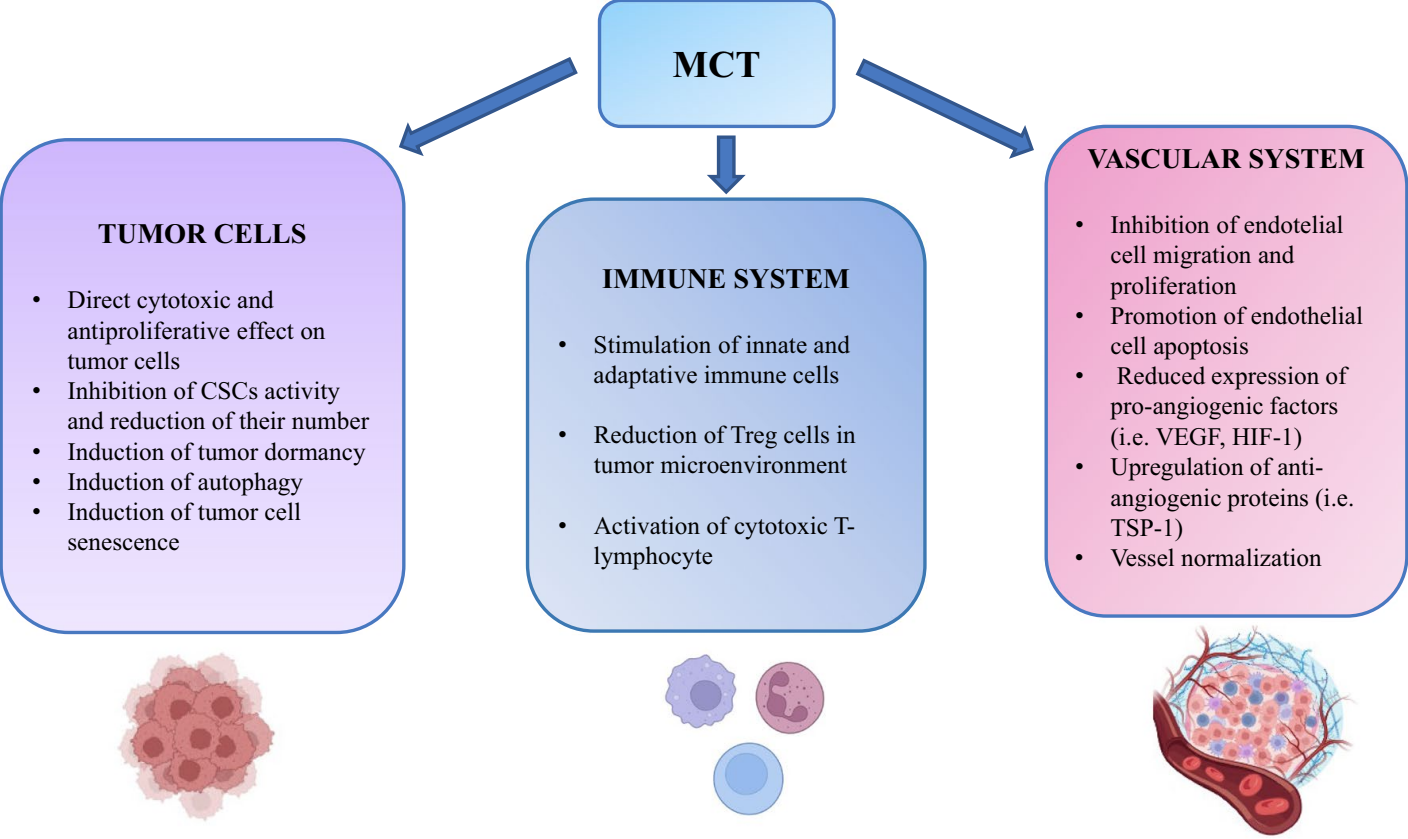
Effects of metronomic chemotherapy (MCT) on cancer cells, immune and vascular system. *CSC*, cancer stem cell; *HIF-1*, hypoxia-inducible factor-1; *Treg*, regularity T cell; *TSP-1*, thrombospondin-1; *VEGF*, vascular endothelial growth factor

Preclinical and clinical research has elucidated the mechanisms behind the antitumor activity in low-dose metronomic chemotherapy regimens. The initial results suggested that MCT was preferentially active on cycling endothelial cells in the tumor’s vascular system, preventing neoplastic angiogenesis. [11, 12]. Subsequently, it was demonstrated that the mechanisms of action of MCT were multiple, and thus MCT might be defined as “multi-targeted therapy”. MCT is cytotoxic for neoplastic cells, is anti-angiogenic, promotes immune response to the tumor, induces dormancy and senescence of neoplastic cells, and may eradicate tumor stem cells. (Fig. 1) [12, 13, 14, 15, 16].

Overall, MCT may offer many therapeutic advantages when compared with conventional MTD schedules (Table 1). These benefits are most likely in the treatment of older people in terms of antitumor efficacy, toxicity, and quality of life preservation [17]. Since MCT schedules are generally built on oral administration of c drugs, they enable old patients to self-administer the drugs at home for all duration of treatment. The lower incidence of adverse drug events also reduces the need of clinic visits and blood tests [18–20].

**Table 1:** Main characteristics of maximum tolerated dose (MTD) and metronomic chemotherapy (MCT)

	MTD	MCT
Dose	Maximum tolerated dose	Low dose empirically conceived (1/10 to 1/3 of MTD)
Administration frequency	Cycles, defined ranges	Frequent or continuous administration without interruption
Pharmacokinetics	High peaks in plasma drug concentration	Low plasma concentration but constant over time
Target	Rapidly dividing cancer cells	Tumor cells, tumor stem cells and angiogenesis
Toxicity	Severe or cumulative toxicity	Low toxicity
Tumor Immunity	Immunosuppressive activity with alteration of antitumor immunological response	Anti-cancer immunity stimulation
Aim of the treatment	Cancer eradication	Cancer control
Quality of life	Reduction	Enhancement

In the beginning MCT has been confined to the palliative setting. In the last decade, given its demonstrable clinical activity MCT has been utilized more and more frequently as front-line treatment for patients who are ineligible for MTD therapy [21].

Although MCT has a good efficacy and it could be used in cancer therapy to overcome the limitations and disadvantages of conventional chemotherapy, the MCT is not completely free from adverse drug reactions (ADR). High-grade toxic effects are infrequent but low-grade fatigue, mild nausea and vomiting, mild to moderate anemia, neutropenia, leukopenia, and lymphopenia, as well as drug-related toxic events, have been documented [15, 22].

A recent study looked at the toxicity profile and adverse drug reactions in geriatric cancer patients. A hundred twenty-nine individuals, 65 years of age and older, who had late-stage cancer, received MCT as front-line treatment. The only demonstrable toxicity included grade 1 anemia. No adverse drug interactions were detected. [23, 24•].

MCT has been tested in several clinical trials to explore effectiveness and safety in geriatrics patients with different types of tumors. In this mini review we describe and comment the most relevant studies of the scientific literature regarding MCT used for the most common cancers involving the geriatric population.

## Metronomic Chemotherapy in Elderly Breast Cancer Patients

Breast cancer is the most common cancer in older women to be diagnosed and a major cause of deaths [25]. Unfortunately, standard treatments are not always suitable for elderly women, and undertreatment, may result in reduced survival. For this reason, MCT may be a valid alternative in this patient’s group (Table 2) [26, 27].

**Table 2:** Metronomic clinical studies that have been performed in elderly breast cancer. For abbreviations, see the text of the article

Tumor type	Number of patients	Metronomic drug	Age	Activity	Toxicity	Ref
Metastatic breast cancer	34	Vinorelbine	Median age 74 years (range 70–84)	CR: 6%; PR: 32%: median PFS 7.7 months; median OS 15.9 months	Not reported	Addeo et al. [31]
Metastatic breast cancer	32	Vinorelbine	≥ 70 years old; median age 76 years (range 69–83)	ORR: 68.7%, including CR: 18% and PR: 50%; DCR 87.4% in 18.7% of patients; median PFS 9.2 months	Not reported	De Iuliis et al. [28]
Locally advanced or metastatic breast cancer	32	Vinorelbine Capecitabine	Median age 76 years	ORR: 33%; clinical benefit (CR + PR + stable disease for > 24 weeks) achieved in the 67% of patients	127 adverse events of any grade reported. 45.8% of grade 1–2 events (7.9% grade 2 asthenia, 6.1% grade 1 nausea, 5.3% grade 1 asthenia, 4.9% grade 1 abdominal pain and 2.3% diarrhea). Very low incidence of grade 3 (1.5%) and grade 4 (0.7%) adverse events (0.7% grade 3 neuropathy and 0.7% grade 4 neutropenia)	Cazzaniga et al. [32]
HER2 + metastatic breast cancer	80	Cyclophosphamide	> 60 and > 70 years old	Median PFS (at median follow-up of 20.7 months): 5.6 months with trastuzumab and pertuzumab vs 12.7 months with the addition of oral MCT cyclophosphamide	Grade 3–4 adverse events: hypertension (15% in trastuzumab and pertuzumab group vs 12% in MCT group), diarrhea (10% vs 12%), dyspnea (5% vs 10%), fatigue (8% vs 5%), pain (5% vs 5%), thromboembolic event (0% vs 10%): severe cardiac toxicities occasionally in both groups	Wildiers et al. [29•]
Endocrine-responsive clinical T2-4 N0-1 breast cancer	144	Cyclophosphamide	> 70 years old or between 65 and 70 years, ineligibles for chemotherapy	ORR: 71.9%; significantly increased suppression of Ki67 and VEGF-A expression in the letrozole/cyclophosphamide-treated group compared with the letrozole-treated group, decreased expression of Ki67 and VEGF in post-treatment residual histology	Most frequent cardiac and bone-related major adverse events: 3 patients fatal heart failure; 1 patient reversible atrial flutter; 3 patients skeletal fractures leading to death in one patient; 3 patients suffered from osteoporotic bone pain. Other adverse events: deep vein thrombosis, mild asthenia (grade 1 and 2) and mental impairment	Bottini et al. [33]
Non-endocrine responsive breast cancer	77	Cyclophosphamide	≥ 66 years old; median age 74 years (range 66–84)	At a median follow-up of 42 months, 81% of randomized patients remained free of any breast cancer recurrence	97% of patients experienced adverse event: 51% of cases in PLD group while 34% in CM group grade 3 adverse events (8 cases of hand-foot skin reaction and 7 cases of hypertension at CM). No grade 4 or higher adverse events reported	Crivellari et al. [34]

Several phase II trials studied oral vinorelbine (VNR) as single agent or in combination with capecitabine in older women with metastatic breast cancer. Clinical benefits were obtained in the majority of patients and a number of complete responses were achieved. No treatment related death was reported, and the toxicity appeared manageable [28, 29•, 30–32].

Of special interest is the use of MCT with cyclophosphamide. Indeed, metronomic cyclophosphamide added to trastuzumab plus pertuzumab (TP) combination resulted in longer PFS (12.7 months) compared to TP administered alone (5.6 months) in a phase II study of elderly patients with HER2 + metastatic breast cancer, aged 65 to 70 [29•].

Bottini and colleagues conduced a randomized phase II trial enrolling 144 elderly patients over 70 years of age and not eligible for chemotherapy with endocrine-responsive clinical. All patients had T2-4 N0-1 breast cancer and were randomly assigned to receive either letrozole alone or letrozole plus metronomic cyclophosphamide as a preoperative treatment for six months. The ORR for the letrozole alone-treated group was 71.9% (95% CI 60.0–83.8%) whereas it was 87.7% (95% CI 78.6–96.2%) for the group treated with letrozole plus metronomic cyclophosphamide. The combination therapy significantly reduced the histological expression of vascular endothelial growth factor-A (VEGF-A) and Ki67. Adverse events were similar in both groups [33].

The International Breast Cancer Study Group performed the multinational randomized phase III clinical trial CASA (Chemotherapy Adjuvant Study for women at advanced age) to compare pegylated liposomal doxorubicin every two weeks with MCT in women 66 and olde with operable, non-endocrine responsive breast cancer [34]. MCT consisted of daily cyclophosphamide (50 mg) and twice weekly oral methotrexate (2.5 mg). The two regimens had comparable activity, but MTC was associated with better quality of life and cognitive function.

## Metronomic Chemotherapy in Elderly Prostate Cancer Patients

Prostate cancer is the second commonest cancer in men, and its occurrence is steadily rising particularly in men over 65 [35]. The risk of metastatic disease at presentation increases with age and is highest over age 80 [35, 36]. Prostate cancer treatment is challenging because many chemotherapy regimens are poorly tolerated by older patients [37]. Androgen Deprivation Therapy, including Androgen Receptor Signaling inhibitors (ARSi) such as abiraterone, apalutamide or enzalutamide, is the gold standard in treating metastatic castration resistant prostate cancer (mCRPC), in combination with either docetaxel chemotherapy (administered three times weekly) and corticosteroids. Though docetaxel is able to keep the tumor under control in many patients with increased survival, its administration at the MTD is associated with short-and long-term adverse drug reactions such as myelosuppression, mucositis, peripheral neuropathy MCT is a safe and effective treatment option that has repeatedly been tested in metastatic prostate cancer patients and might also be considered even in condition where the use of ARSi is not affordable (Table 3) [36–39].

**Table 3:** Metronomic clinical studies that have been performed in elderly prostate cancer patients. For abbreviations, see the text of the article

Tumor type	Number of patients	Metronomic drugs	Age	Activity	Toxicity	Ref
Advanced castration-resistant prostate cancer	29	Cyclophosphamide	≥ 78 years old; median age 83 years (range 78–92)	At a median follow-up of 27.3 months, the median PFS 7.7 months and the median OS 19.7 months; The 62% of patients had a decrease in PSA levels from 2 to 99%, with a median duration of response of 8.6 months	Not reported grade 3 or 4 hematologic or nonhematologic toxicity. 14% of patients reported grade 2 anemia, 7% developed grade 2 thrombocytopenia. No toxicity-related major cardiovascular events or deaths were observed	Fontana et al. [40•]
Metastatic castration-resistant prostate cancer	26	Vinorelbine	Median age 78.1 years (range 70–87)	No statistical difference between the two groups for PFS at 6 months (57.1% for mVNR group vs 58.3% for docetaxel group); PFS: 8.6 months for mVNR group and 8.2 months for docetaxel group	The most frequent side effect was anemia between mVNR and docetaxel group (8% vs. 7% Grade 3) and vomiting (5% Grade 3 vs. 2%,); grade 3 constipation (5% vs. 0%). Severe vomiting in 5% of patients treated with metronomic VNR	Tralongo et al.[43]

In a retrospective study, 29 elderly patients aged 78 years or older affected by CRPC with several comorbidities and geriatric syndromes, 65% of whom were described as “frail”, received 50 mg daily of oral metronomic cyclophosphamide in combination with celecoxib 200 mg twice a day and 1 mg of dexamethasone for a period of 12 weeks. The results showed that 18 patients (62%) had a decrease in Prostate Specific Antigen (PSA) levels from 2 to 99%.. The median duration of PSA response was 8.6 months (95% CI 7.6–9.6 months). After 27.3 months (95% CI 18.8–35.8 months) of observation, the average median PFS and median OS were 7.7 months (95% CI 2.3–13.1 months) and 19.7 months (95% CI 12.8–26.6 months), respectively [40•]. Daily metronomic cyclophosphamide is renowned for its antiangiogenic properties. In prostate cancer this activity was confirmed with the study of pharmacodynamic markers such as plasma vascular endothelial growth factor levels and vascular endothelial cadherin expression [41]. Furthermore, the cancer progression may be limited due to the ability of metronomic cyclophosphamide to promote immune response to the tumor [42].

In a study involving 26 elderly and vulnerable patients with mCRPC, Tralongo and co-workers compared effectiveness and toxicity of weekly parenteral docetaxel and oral metronomic VNR [43]. There was no statistical difference between the two groups in terms of PFS at 6 months (57.1% for patients receiving VNR versus 58.3% for those who were treated with docetaxel), whereas the PFS for patients treated with oral metronomic vinorelbine and docetaxel was 8.6 months (95% CI 7.1–9.4 months) and 8.2 months (95% CI 6.9–9.3 months), respectively. Anemia was the most common side effect of both drugs whereas the prevalence of vomiting (5% Grade 3 vs. 2%, respectively) was comparable to that of patients receiving docetaxel.

## Metronomic Chemotherapy in Elderly Non-Small Cell Lung Cancer (NSCLC) Patients

Around 30–40% of new cases of advanced NSCLC are detected in population older than 70 years and nearly 15% of cases afflict people aged ≥ 80 years. The vast majority of these patients at the time of diagnosis, are inoperable [44, 45]. Based on the characteristics of the subjects and of the disease, it can be opted between a curative or a palliative approach.

To date, the European Society of Medical Oncology defines monochemotherapy (generally with gemcitabine, vinorelbine and docetaxel) and polychemotherapy (commonly vinorelbine plus cisplatin, but also chemotherapeutic agents in combination with immunotherapy or target therapy) the main therapeutic options available for these elderly patients: polychemotherapy obtains the best results, but has higher risk of toxicity, while single-agent therapy is better tolerated by elderly subjects [46, 47].

In this context, MCT might be used as an alternative therapeutic strategy for elderly and debilitated patients (Table 4) [37]. Indeed, a growing amount of data from preclinical and clinical research studies indicates that oral etoposide and vinorelbine administered in metronomic regimens may be the most promising therapeutic options for elderly patients who are ineligible for MTD therapies [37, 48, 49].

**Table 4:** Metronomic clinical studies that have been performed in elderly NSCLC patients. For abbreviations, see the text of the article

Tumor type	Number of patients	Metronomic drug	Age	Activity	Toxicity	Ref
Advanced non-small cell lung cancer	92	Vinorelbine	≥ 70 years old; median age 81 years (range 68–89)	Median length of therapy: 15 weeks; OS: 32.2 weeks; 60% of patients experienced clinical benefit	4 cases of G4 neutropenia and 5 nonhematologic toxicities (2 asthenia, 2 arthralgia, 1 cutaneous); 8 patients required dose reductions due to transient neutropenia or various symptoms, mainly clinical deterioration/asthenia	Pasini et al. [52]
Advanced non-small cell lung cancer	270	Vinorelbine	Median age 76 years (range 48–92)	ORR: 17.8% 46 PR and 2 CR); 44.1% of patients experienced stable disease > 12 weeks; DCR of 61.9%	2% of grade 3/4 toxicity (mainly G3 fatigue and anemia) and no toxic deaths	Camerini et al. [47]
Advanced non-small cell lung cancer	44	Vinorelbine	> 60 years old; median age 77 years	DCR: 63%. Median PFS: 9 months Median OS: 12 months Evidence of disease progression in 36% of patients	Not reported	D’Ascanio et al. [53•]
Advanced non-small cell lung cancer	293	Vinorelbine	Median age 76 years (range 39–94), of which 71% ≥ 70 years	The ORR: 18%, with 42 partial responses and 0 complete responses; DCR: 72%.; 54% of patients manifested stable disease and 28% of patients manifested disease progression	The 46% of patients experienced grade 1/2 toxicity, including 31.1% G1/2 fatigue, 30.7% G1/2 anemia, and 15.0% G1/2 nausea or vomiting 21% of patients reported grade 3/4 toxicity, 10.2% G3/4 neutropenia and 9.6% G3 fatigue; 33% of patients experienced no toxicity with mVNR	Estevinho et al. [48]

VNR was the first compound investigated as metronomic treatment in the elderly patients affected by NSCLC [47, 50]. Metronomic oral vinorelbine (MOV) is also listed by The Geriatric Oncology Working Group of the Spanish Society of Medical Oncology in its 2018 guidelines as an appropriate alternative to handle elderly patients affected by NSCLC [51].

Pasini and colleagues demonstrated that the use of MOV resulted in important survival in the elderly or pre-treated patients (mean age 81). Forty percent of these patients had received two different treatment regimens prior to MOV. They also assessed the blood concentration of the drugs and found it stable for a long period of time. Mean VNR values were 3.2 vs. 1.9 nM (*P* = 0.012), and VNR + 4-O-deacetylVNR (active metabolite of vinorelbine) were 7.5 vs. 3.7 nM, respectively (*P* = 0.032). To achieve a more constant systemic exposure to VNR and avoid high peak values, single doses were diminished from the traditional oral 50 mg to 20–30 mg and were given every two days without pause. At the end of the study, the median length of therapy and overall survival were 15 and 32.3 weeks, respectively [52].

Both safety and efficacy of MOV were also demonstrated in a multicenter, global retrospective study from 270 patients, with a median age of 76. Patients received an overall average of 6 cycles of MOV consisting of 50 mg, 40 mg, 30 mg, three times weekly continuously. The study confirmed that MOV was safe in the first and subsequent lines of treatment, with an interesting activity in long-term disease stabilization without worsening its toxicity profile [47].

In another study, 44 cases with median age of 77 years, affected by NSCLC at IIIB or IV stage and at least one major organ disfunction including renal, hepatic, or cardiac disorder, were enrolled between January and December 2016 [53•]. Patients were treated with 30 or 40 mg of oral metronomic VNR thrice a week. VNR showed an improvement in patient’s compliance (rate of 85%), defined by the consumption of more than 75% of the recommended prescription; in addition, VNR presented a satisfactory safety profile and demonstrated efficacy in slowing cancer progression. The DCR was 63%. In 36% of the individuals, there was evidence of disease progression. Nine months was the median PFS, while 12 months was the median overall survival (OS). When comparing the two program types (30 vs. 40 mg × 3/week), no statistically significant results (HR = 1.1) were identified. On the other hand, an HR of 0.72 (*P* = 0.006) indicated a statistically significant difference in PFS depending on age (more or less than 75 years).

A multicenter retrospective study was conducted between 2016 and 2018 assessing MOV schedule in 19 cancer centers in Portugal [48]. The researchers enrolled 293 patients, with an average age of 76. Among these patients, 42% underwent oral metronomic VNR as first-line therapy, with a follow-up of 4 months. While 28% of patients had a progression of the disease, 18% of patients experienced a response and 54% of subjects had stable disease. Only 5% of patients had treatment discontinuation due to documented adverse events.

## Metronomic Chemotherapy in Elderly Non-Hodgkin Lymphomas Patients

MCT was studied in different forms of NHL including B and T cell Lymphomas (Table 5).

**Table 5:** Metronomic clinical studies that have been performed in elderly NHL patients. For abbreviations, see the text of the article

Tumor type	Number of patients	Metronomic drug	Age	Activity	Toxicity	Ref
Relapsed or refractory non-Hodgkin’s Lymphoma	50	Prednisone, etoposide and cyclophosphamide	Range 20–80 years old	Test group ORR: 30.4% (1 CR and 6 PR), DCR: 87.0% (1 CR, 6 PR and 13 SD). Control group ORR: 38.1% (2 CR and 6 PR), DCR: 57.1% (2 CR, 6 PR and 4 SD) During the 12-month follow-up, test group ORR: 47.8% (3 CR and 8 PR), DCR: 69.6% (3 CR, 8 PR and 5 SD). ORR control group: 19.0% (1 and CR and 3 PR), DCR:33.3% (1 CR, 3 PR and 3 SD)	No grade 3/4/5 adverse effects and no toxicity-related deaths reported in either group	Zeng et al. [61]
Diffuse large B- and T- cell lymphomas	55	DEVEC	Median age 80 years old (range 56–93)	Median OS: 13 months; PFS:11 months	22.2% of G ≥ 3 extra hematological adverse events (7 neutropenic fever or infection requiring hospitalization, 1 pulmonary embolism, 1 heart failure, and 1 multi organ failure); 1 of these patients died; 4 discontinued treatment During the induction phase, 60% of patients had neutropenia ≥ G3 and 4.4% had anemia ≥ G3	Cox et al.[57••]
Diffuse large B-cell Lymphoma	51	DEVEC	Frail ≥ 65 years or ineligible ≥ 85 years; R/R ≥ 55 years; median age 85 years naive patients (range 77–93), R/R patients 78 years (range 57–91)	OS and PFS at one year were 67% and 61% for DEVEC-naïve, 60% and 50% for reference-naïve; OS, PFS and FFS at one year: 67%, 61% and 55% for DEVEC-naïve, 60%, 50% and 50% for reference-naïve, respectively	43% hematologic adverse events of G ≥ 3 (G3 neutropenia was the most frequent); 13.7% extra hematologic adverse events of grade ≥ 3 (infection was the most frequent). 5.9% severe hematologic toxicity events (grade 4 cytopenia lasting more than 6 days) in heavily pretreated patients or those with bone marrow involvement	Cox et al.[65]
Diffuse large B-cell Lymphoma	22	DEVEC	Mean age 84.5 years	At the mid-term evaluation, ORR and CRR were 77% and 32%, respectively, while at the end of induction the ORR and CRR were both 64%. OS and EFS at 24 months were both 54%, while DFS 74%	27% of patients experienced treatment-related serious adverse events (3 febrile neutropenia, 1 urosepsis and 2 cases of pneumonia)	Bocci et al. [66••]
Peripheral T cell Lymphoma	17	DEVEC	Median age 83 years (range 71–83)	ORR: 80% naïve group, 58% RR group. CR observed in 20% naive and 25% RR. Median PFS naïve: 20 months; median OS naïve: 46 months Median OS R/R: 13 months; median PFS R/R: 11 months	TRAE: 47% (35% of G3 neutropenia). 4 G4 neutropenia lasting more than 6 days occurred in 17.6% of patients who had been heavily pretreated or with bone marrow involvement. 5 non-hematological. TRAEs of grade ≥ 3 (1 bacterial meningitis, 2 pneumonia, 2 neutropenic sepsis) were recorded in 23.5% patients	Cox et al.[55•]
Peripheral T cell Lymphoma	12	TEPIP	Median age 70 years	ORR: 42% (CR 25%); OS averaged 185 days	Any AE grade occurred in 8 of 12 patients, mainly nonhematological	Fante et al.[67]
Recurrent mantle cell Lymphoma	25	RT-PEPC	Median age 68 years (range 52–81)	At a median follow-up of 38 months, ORR: 73% (CR/unconfirmed CR rate, 32%; PR rate, 41%); median PFS response rate: 10 months Decrease of VEGF plasma levels and a decreasing trend of CECs	Toxicities included grade 1 and 2 fatigue, rash, neuropathy and cytopenia, including 64% of grade 1 and 2 thrombocytopenia and 64% of grade 3 and 4 neutropenia; 2 thromboses and 5 episodes of grade 3 or 4 infections occurred	Ruan et al. [68]

The most common treatment of Large Cell B Cell Non-Hodgkin lymphomas (NHL) includes the bination of chemotherapy (cyclophosphamide, doxorubicin, vincristine, and prednisolone, CHOP) and the monoclonal antibody Rituximab [54]. This regimen has been studied in individuals 60 and older with high remission rate. Nonetheless vulnerable and frail older patients may not tolerate this regimen, even with the addition of hematopoietic growth factors. The inability to administer CHOP-rituximab in full doses has led to lesser and shorter remissions. [55•, 56, 57••]. MCT has been explored as an alternative treatment in these patients [58]. The limited available data suggest that MCT with cyclophosphamide, trofosfamide and etoposide, has significant activity *(*Table 5*)* [59–62]. It is important to underline that Peyrade et al. demonstrated that reduced doses of CHOP in combination with rituximab or ofatumumab were active and well tolerated in individual ≥ 80 [63, 64]. Any randomized study of MCT in older and frail patients should include these dose-reduced regimens of CHOP as standard treatment.

In 2008 Coleman et al. studied the activity of MCT with PEP-C (cyclophosphamide, etoposide, procarbazine, and prednisolone) in low grade lymphoma [62]. PEP-C therapy yielded 36% CR and 33% PR. The treatment regimen was generally well tolerated.

A randomized controlled phase II trial enrolling subjects with aggressive B-cell subtypes showed that a fully oral metronomic schedule built on cyclophosphamide, etoposide, and prednisolone resulted in comparable response rates to the standard treatment scheme [61]. This treatment schedule was empirically designed as a palliative therapy using a combination of drugs whose activity was already known in NHL even in monotherapy i.e. (prednisone, etoposide, vinorelbine, cyclophosphamide). Circulating endothelial cells (CECs) and VEGF serum levels as possible biomarkers for this therapeutic approach were two of the study's primary endpoints. Serum levels of VEGF and number of CECs in the metronomic group were considerably lower after two treatment cycles than in the control group. (*P* < 0.05). The metronomic group's DCR (87.0%) was significantly higher than the control group's (57.1%) after two treatment cycles. The ORR and DCR (47.8 and 69.6%, respectively) were greater in the metronomic-treated patients if compared to the control group's (19.0 and 33.3, respectively) after 12 months of therapy. PFS was significantly longer in the group treated with MCT, whereas the toxicity was significantly reduced. Patients in the metronomic arm had a median PFS of 14 months (95% CI: 11.2–16.7 months), whereas those in the control arm had a median PFS of 7.5 months (95% CI: 4.9–10.1 months) with a P of 0.004 [61].

In 2011 it was demonstrated that a novel oral MCT scheme known as DEVEC (Deltacortene®, etoposide, vinorelbine, cyclophosphamide) could be safely administered in patients with DLBLC unfit for standard MTD [57••]. The DEVEC protocol involves consecutive cycles of therapy with short drug-free breaks that allow hematologic recovery and the maintenance of prolonged exposure to various chemotherapeutic drugs throughout the cycle, thereby preventing the appearance of chemoresistance. DEVEC has been demonstrated to be effective for palliative treatment of elderly patients with DLBCL by a multicentric retrospective study involving 6 Italian centers since March 2011 [65]. DEVEC was initially administered to 51 patients with DLBCL not eligible for intravenous MTD chemotherapy regimens, of whom 33% were treatment naïve (mean age 85 years) and 67% were R/R (mean 78 years). The study’s findings showed that DEVEC caused CRs and enabled long-term remissions in both groups. While more clinical research is required to properly assess the advantages of oral DEVEC over conventional intravenous protocols, this low-cost program appears to be ideal for elderly or fragile patients who need to minimize hospitalization and reduce toxicity with customized treatments.

A multicentric observational study gathered data from six clinical centers using the oral DEVEC metronomic program for the treatment of 22 elderly and frail patients (mean age 84.5 years) with DLBCL not considered suitable for treatment with IV-CHEMO [66••]. At the mid-term evaluation, ORR and CRR were 77% and 32% respectively, while at the end of the induction they were both 64%. OS and 24-month event-free survival were both 54% (95% IC = 32–72), while disease free survival was 74% (95% IC = 48–88%). Overall, severe treatment-related adverse events were recorded in 27% of subjects (IC 95% = 14–33), and included three febrile neutropenia, one urosepsis and two cases of pneumonia. This observation positively underlines the tolerability and activity of R-DEVEC. However, to avoid excessive toxicity, etoposide should be eliminated in the most debilitated subjects, who may be safely treated with the combination Rituximab, VNR, cyclophosphamide and prednisolone (R-DEVEC-light). Interestingly, 50% patients treated with R-DEVEC-light reached CR and had prolonged remission after maintenance cycles. Conversely, maintenance may not be necessary in naïve patients at treatment who reach CR after six cycles of R-DEVEC [66••].

Indeed, DEVEC oral metronomic schedule can also be successfully used in elderly patients with T-cell lymphoma. Four Italian clinical centers prospectively collected data on 17 elderly patients with peripheral T-cell lymphoma (PTCL) treated with DEVEC metronomic scheme. Of these 29.4% were treatment-naïve (mean age 83 years), while the remaining 70.6% were refractory to the treatment or relapsed (mean age 71.5 years), but all were classified as frail [55•]. The metronomic schedule was planned with an induction phase and a de-escalated maintenance phase, both consisting of 6 cycles lasting 28 days. In subjects who achieved less than CR, maintenance cycles were administered alternating: cyclophosphamide 50 mg for 14 days/etoposide 50 mg for 7 days and cyclophosphamide 50 mg for 14 days/ vinorelbine 30 mg three times a week 3 weeks yes/1 week no, until progression or excessive toxicity. The oral DEVEC therapy showed encouraging activity and acceptable toxicity: hematologic treatment-related adverse events (TRAEs) were recorded in 8 patients, the most frequent being G3 neutropenia in 35% of subjects. A total of 23.5% of patients reported non-hematologic TRAEs of grade ≥ 3, while no treatment-related deaths. Overall, the 47% of patients had etoposide dose reductions. The median follow-up, from the start of treatment, was 45 months (range 14–72). Tumor reduction was seen in all (100%) naïve patients (95% CI 55–100%) and in 75% of RRs (95% CI 43–95%). At the end of the induction phase, the ORR was 80 and 58% in the naïve and RR patients, respectively. CR was observed in 20% of naïve and 25% of RR subjects, respectively. The median PFS and OS for naïve patients were 20 (95% CI 0–43) and 46 months, respectively, whereas in RR individuals, PFS was 11 months (95% CI 4.2–17.8) and OS was 13 months (95% CI 11.3–14.6).

In a retrospective, single-center observational study, the safety and efficacy of TEPIP (including trofosfamide, etoposide, procarbazine, idarubicine and prednisolone) were evaluated in 12 elderly patients (median 70 years, extensive disease and poor prognosis) with PTCL and treated at Regensburg University Medical Center between 2010 and 2022 [67]. The endpoints were overall ORR and OS. TEPIP was administered as an oral-only chemotherapy regimen allowing completely outpatient treatment, including: 50 mg of trofosfamide, 50 mg of etoposide, 100 mg of procarbazine, and prednisolone at 100 mg on days 1 to 10, which was reduced to 7 days in case of numerous pretreatments or advanced age (> 65 years). The cycle was repeated every 28 days provided the leukocyte count exceeded 3,000/μl and continued until disease progression or adverse events occurred. After an average of 2.5 TEPIP cycles (total of 83 cycles), ORR was 42%, CR 25% and OS averaged 185 days. Any grade of adverse event occurred in 8 of 12 patients, with four patients showing adverse events ≥ CTCAE grade 3 (33%), and adverse events were mainly not hematologic. Three patients, including one heavily pretreated patient with relapse from high-dose chemotherapy and subsequent autologous stem cell transplantation achieved complete remission (25%) in response to TEPIP treatment. In addition, two patients showed transient partial remissions of T-cell lymphoma lesions, and stable disease was observed in two other patients. To sum up, all-oral TEPIP treatment conducted in the outpatient setting has the potential for durable remissions in heavily pretreated patients with advanced T-cell lymphoma [67].

In a retrospective analysis, 25 elderly patients (mean age 68 years) with recurrent mantle cell non-Hodgkin's lymphoma (MCL) received metronomic RT-PEPC consisting of prednisone (20 mg), etoposide (50 mg), procarbazine (50 mg), and cyclophosphamide (50 mg) administered orally on a continuous schedule also including administration (months 1–3) of rituximab 4 times a week, daily thalidomide (50 mg) followed by maintenance thalidomide (100 mg). Study endpoints included safety, efficacy, quality of life and translational studies, including tumor angiogenic phenotyping, VEGF and CECs [68]. RT-PEPC showed significant and durable activity in MCL with manageable toxicity and maintained good quality of life. At a median follow-up of 38 months the 2-year PFS rate was 24% (95% CI, 10%-56%) and the median PFS was 10 months (95% CI, 5–23 months), whereas the 2-year OS rate was 45% (95% CI, 28%-76%); the ORR was 73% (95% CI, 50%-89%), with an CR/CRu rate of 32% and the PR rate was 41%. It has also been observed a decrease of VEGF plasma levels and a decreasing trend of CECs. Response (CR/PR) to treatment with RT-PEPC was favorably associated with PFS (HR, 0.13; 95% CI, 0.04–0.48; *P* < 0.002). The therapy was generally well tolerated: nonhematologic toxicities were mostly mild and comprised grade 1 or 2 events such as rash, fatigue, diarrhea, cough, nausea, neuropathy, and dyspnea, whereas hematologic toxicities included anemia as well as other grade 3 or 4 events.

## Metronomic Chemotherapy in Elderly Patients With Different Tumor Types

From published data, it is possible to observe that MCT has been successfully used in elderly patients also in other types of cancer (Table 6).

**Table 6:** Metronomic clinical studies and case reports that have been performed in elderly patients with different tumor types. For abbreviations, see the text of the article

Tumor type	Number of patients	Metronomic drug	Age	Activity	Toxicity	Ref
Metastatic melanoma	13	Cyclophosphamide	≥ 65 years old; median age 80 years (range 70–92)	CR confirmed in 1 case, SD confirmed in 5 cases (median duration: 10 months) and progressive disease in five cases. The control rate at 2 months: 46% (1 PR and 5 SD) OS in patients with stage III and IV MM: 6 to 36 months and 4 to 24 months, respectively At the end of the study, 23% of the patients were still alive, with follow-up of 15, 25 and 36 months from the start of treatment	Interruption of treatment for 3 patients because of malaise and cough, pneumonia, pancytopenia; temporary suspension of treatment because of abdominal discomfort for 1 patient, erysipelas for 3 patients All biological adverse events, with the exception of preexisting lymphopenia, were reversible when the dose of cyclophosphamide was reduced or when treatment was interrupted	Borne et al. [69]
Ovarian cancer	16	Cyclophosphamide	DL1: median age 63 years (range 57–73); DL2: median age 66 years (range 54–73); DL3: median age 70.5 years (range 68–74)	Median PFS: 8.35 months; median OS: 24.95 months 50% of treated patients showed CR or PR after 12 weeks at DL I, 20% at DL II and 33% at DL III	Total of 155 AEs: 87.5% increased AST and 75% ALT, 50% diarrhea, 37.5% leukopenia, 37.5% fatigue 62.5% of the patients died, 80% of them due to disease progression Hypertension was the only AEs related to the experimental drug	Dinkic et al. [70]
Advanced gastric cancer	45	Capecitabine	> 70 years old; median age 74.5 years (range 71–81)	PR achieved in 20.9% of patients; SD achieved in 30.2%; 48.8% had disease progression DCR: 51.1% Median TTP: 3.6 months; median OS: 7.6 months; 1-year survival rate: 28.5%	Grade III neutropenia in 8.9% of patients; grade II and III hand-foot syndrome and stomatitis in 15.5% of patients 20% of patients experienced treatment delays due to hematologic adverse events (8.9%), hand-foot syndrome (2.2%), anorexia (2.2%) and stomatitis (6.7%)	He et al. [71]
Metastatic colon cancer	1	Capecitabine	82 years old	CR reached at the completed of the cycles; until the last follow-up, the disease remained CR PFS: about 87 months	Grade 1 leukopenia and mild, controllable hand-foot syndrome	Liu et al. [72]
Metastatic squamous cell carcinoma	1	Capecitabine	66 years old	Disease control for further 18 months with metronomic capcitabine; both body weight and oral food consumption did not change during the treatment	Low-grade stomatitis	Muratori et al.[73]
Acute myeloid leukemia	81	Etoposide	≥ 55 years old;	Median survival time: 130 days (MCT groups) vs 70 days (palliative group)	Not reported	Phinyo et al. [74]
Metastatic soft-tissue sarcoma	120	Trofosfamide	≥ 60 years old; median age 70 years (range 60–89)	Median follow-up: 10.4 months for all patients and 18.4 months for patients who survived (arm A: 26 months; arm B: 15.2 months) Objective response rate was 7.7% (1.6–20.9) in arm A (no CR); 3 PR) and 6.6% in arm B (2 CR; 3 PR); DCR (including SD) was 53.8% and 40.8%; PFS was 4.3 months and 2.8 months; OS was 9.8 months and 12.3 months	The 10% of patients were treated with TRO for more than 1 year, showing a favorable toxicity profile that allows long-term treatment application. Safety analyses in 115 patients revealed at least one side effect in 97.4% vs 96.1% of patients Serious adverse effects (grade III or IV) were lower in arm B (59% vs 30.3%). TRO was associated with dyspnea and fatigue (grade I/II), DOX with leukocytopenia, neutropenia and mucositis The all-cause mortality rate within 60 days of drug initiation was 7.7% and 8.0%, respectively	Hartmann et al. [75]

In a phase II clinical trial, elderly melanoma patients treated with metronomic cyclophosphamide demonstrated a response rate and survival values comparable to those seen with traditional chemotherapy with dacarbazine [69]. The study included 10 women and 3 men, with a median age of 80 years. During the first cycle (one month), patients received a daily oral dose of 50 mg metronomic cyclophosphamide. In subsequent cycles, the dose was adjusted to 100 mg cyclophosphamide once daily for three weeks out of four. One patient had a verified PR, whereas five cases had confirmed SD (median duration: 10 months). Patients with stage III and stage IV metastatic melanoma had OS in the range of 6 to 36 months and 4 to 24 months, respectively [69].

In a multicenter phase I trial, 16 elderly patients with platinum-resistant recurrent epithelial ovarian cancer were treated with pazopanib and metronomic cyclophosphamide [70]. The study assessed the combination's best dose, and tolerability. Cyclophosphamide 50 mg was taken orally each day in association with 400–800 mg of pazopanib. One patient out of six experienced dose-limiting toxicity (DLT) at dosage levels (DL) I and II. Two of the four patients at DL III demonstrated DLT, leading to a 600 mg/day MTD of pazopanib. 3 patients received treatment for at least 13 months, with a median of 6 (2–13) cycles being delivered. The median PFS was 8.35 months, while the median OS was 24.95 months. The adverse events included leukopenia, tiredness, diarrhea, and elevated liver enzymes [70].

He and colleagues assessed the efficacy and safety of capecitabine as a palliative treatment administered at a metronomic dose of 1 mg per day to 45 elderly patients (mean age 74.5 years) with advanced gastric cancer (including 33 men and 12 women) who had previously been treated with fluoropyrimidine chemotherapy [71]. The 8-week DCR of metronomic treatment was 51.1% (95% CI 25.7–67.8), and 20.9% (95% CI 13.1–38.5) of the 43 evaluable patients showed an overall response. Age, sex, weight loss, performance status, the presence of liver, peritoneal, lymph node or bone metastases, surgery, and response to previous first- or second-line chemotherapy did not significantly alter the response rate. The study found that the median time to progression (TTP) was 3.6 months (95% CI: 3.2–4.0 months) and the median OS was 7.6 months (95% CI: 7.0–8.2 months). There were no grade IV toxicities, neutropenic fever, or treatment-related fatalities, and only 13.3% and 2.2% of patients, respectively, showed signs of grade II neutropenia and thrombocytopenia [71].

In 2009, a case report described that the use of metronomic capecitabine as a first line and maintenance treatment for an elderly patient with advanced or metastatic colorectal cancer [72]. The 82-year-old patient was treated with capecitabine (1250 mg/m^2^) twice a day for days 1 through 14, every three weeks, for a total of 12 cycles. An additional 4 cycles were then administered as maintenance therapy. After eight cycles, the patient was in CR and capecitabine was continued as a maintenance medication. The disease was still in CR at the time of the final follow-up, and the PFS was roughly 87 months. After two cycles, hepatic metastases decreased by 20% and vanished entirely after eight cycles. Interestingly, the patient experienced hand-foot syndrome and mild, manageable grade 1 leukopenia during the treatment, but there was no sign of metastasis or recurrence [72].

Another interesting use of metronomic capecitabine was reported in the case of an elderly man (66 years old) with metastatic oral cancer. The patient had to stop maintenance with cetuximab (250 mg/m^2^ each week) but achieved prolonged disease control with daily metronomic capecitabine (2,000 mg/day) [73]. In this subject, daily metronomic capecitabine produced an extra 18 months of disease management without clinically significant side effects. The patient only experienced low-grade stomatitis, he did not have diarrhea or palmoplantar erythrodysesthesia. Furthermore, both body weight and oral food consumption did not change during the course of the medication.

Between December 2014 and December 2017, a multicenter randomized and controlled clinical trial conducted in Thailand investigated whether MCT had a time-dependent effect on the treatment of elderly individuals with acute myeloid leukemia who were unfit for standard chemotherapy [74]. Random assignments were made to treat the included patients with palliative care or MCT. Every three weeks for four cycles, patients in the MCT arm received an oral chemotherapy regimen consisting of 50 mg/m^2^ of etoposide for five days, 60 mg/m^2^ of 6-mercaptopurine, and 40 mg/m^2^ of prednisolone. At the conclusion of the trial, the MCT and palliative therapy groups showed a median survival time of 130 (95% CI 64–115) and 70 (95% CI 41–93) days, respectively [74].

Metronomic trofosfamide was found to be safe in elderly patients (mean age of 70 years) with metastatic soft tissue sarcoma, in an open label, randomized, multicenter phase II-controlled trial [75]. Patients who had not received treatment before, were randomized in two arms: arm A received doxorubicin 60 mg/m^2^ for six cycles, which is the conventional first-line treatment for metastatic soft tissue sarcoma, and arm B received on days 1 through 7, 300 mg of oral trofosfamide daily. The median follow-up period at the conclusion of data collection was 10.4 months (range 0.4–94.7) for all patients and 18.4 months (range 3.8–94.7 months; arm A, 26.0 months; arm B, 15.2 months) for patients who survived [75].

## Conclusions and Future Perspectives

The metronomic approach for the treatment of cancers has clear advantages in terms of reduced adverse drug events, possibility of oral administration, higher quality of life, and low cost, making it widely used in elderly patients whose treatment to date is unsatisfactory. In this review, we outlined clinical trials focused on evaluating MCT as a possible therapeutic alternative in elderly and frail patients, unsuitable for conventional treatment regimens, for the management of various types of cancers, among others breast, prostate, lung, and NH lymphomas.

Although the limitations of published clinical studies must be taken into consideration, including the fact that most of them are not randomized phase II clinical trials and were conducted on a limited number of patients, MCT schemes have proven to be well tolerated, to have a good safety profile and an interesting anti-tumor activity in large percentages of elderly patients enrolled in these studies.

Since MCT is based on oral intake of drugs, it can encourage patient’s adherence to therapy, increasing their quality of life, and diminishing the need for hospital stay, with a consequent reduction in pharmaceutical expenditure.

Currently, as we can see in the Table 7, there are a number of experimental studies underway aimed at testing the use of compounds metronomically administered, either in single or in combination with target therapies well known in clinical practice, for the treatment of a wide range of cancer types, evaluating their efficacy in the adult population, including the elderly.

**Table 7:** Metronomic clinical studies currently recruiting also elderly patients found at clinicaltrials.gov

Clinicaltrials.gov id	Type of study	Tumor	Schedule	Age
NCT05554003	Phase II	Neuroendocrine neoplasms (NENs)	Metronomic oral temozolomide (60 mg/day continuously)	≥ 18 years
NCT04304352	Phase II	Metastatic Breast Cancer	Metronomic oral cyclophosphamide (50 mg/die) + capecitabine (500 mg thrice daily) + vinorelbine (40 mg 3 times a week)	18 to 99 years
NCT04941885	Phase II	Metastatic HER2 + /​HR + Breast Cancer	Inetetamab + metronomic oral cyclophosphamide (50 mg/day) + aromatase inhibitor (once a day orally)	18 to 75 years
NCT05462613	Phase II/III	Metastatic Colorectal Cancer	Regorafenib + metronomic oral capecitabine (625 mg/m^2^/ twice daily) + metronomic cyclophosphamide (50 mg/day for two months) + aspirin (75 mg/day orally and daily for two months) + bevacizumab (5 mg/Kg every 2 weeks + FOLFIRI or FOLFOX	≥ 18 years
NCT06054906	Phase II	Advanced Gastric Cancer	Sintilimab + metronomic PLOF (paclitaxel 60 mg/m^2^ + oxaliplatin 50 mg/m^2^ + 5-fluorouracil 425 mg/m^2^)	≥ 18 years
NCT05044117	Phase III	Advanced Head and Neck Squamous Cell Carcinoma	Capecitabine (650 mg/m^2^) for 1 year	18 to 70 years
NCT03561740	Phase III	High Risk HER2 + Breast Cancer	Metronomic capecitabine	18 to 70 years
NCT04350021	Phase II	Metastatic Breast Cancer	Metronomic capecitabine 500 mg times three, cyclophosphamide 50 mg once daily	≥ 18 years
NCT05063136	Phase III	HR + /​HER2- Primary Breast Cancer	Metronomic capecitabine (500 mg/day for 1 year) + standard endocrine therapy (at least 5 years)	18 to 70 years
NCT05390476	Phase II	Triple Negative Breast Cancer	Tucidinostat + metronomic capecitabine (500 mg orally three times a day)	18 to 75 years

It is desirable for the future, to develop initiatives to extend cooperative clinical trials including only older patients, with comorbidities and frailty, and to broaden the variety of investigated tumor types, in order to support the application of MCT as a possible standard of care in the elderly patients, and not only limited to palliative intent.

## Data Availability

No datasets were generated or analysed during the current study.

## References

[1] Repetto L, Balducci L (2002). A case for geriatric oncology. Lancet Oncol.

[2] Scotté F, Bossi P, Carola E, Cudennec T, Dielenseger P, Gomes F (2018). Addressing the quality of life needs of older patients with cancer: A SIOG consensus paper and practical guide. Ann Oncol.

[3] Kumar Pal S, Katheria V, Hurria A (2010). Evaluating the Older Patient with Cancer: Understanding Frailty and the Geriatric Assessment. CA Cancer J Clin..

[4] Bellelli F, Consorti E, Hettiarachchige TMK, Rossi P, Lucchi T, Froldi M (2023). Relationship among age, education and frailty in older persons. J Frailty Aging..

[5] Clegg A, Young J, Iliffe S, Rikkert MO, Rockwood K (2013). Frailty in elderly people. The Lancet..

[6] Kareva I, Waxman DJ, Klement GL (2015). Metronomic chemotherapy: An attractive alternative to maximum tolerated dose therapy that can activate anti-tumor immunity and minimize therapeutic resistance. Cancer Lett..

[7.••] Tralongo AC, Fratamico RS, Russo C, Sbrana A, Antonuzzo A, Danova M (2021). Anti-cancer treatment strategies in the older population: Time to test more?. Geriatrics.

[8.••] Bocci G, Kerbel RS (2016). Pharmacokinetics of metronomic chemotherapy: A neglected but crucial aspect. Nat Rev Clin Oncol.

[9] Kim J, Kim YM (2019). Tumor endothelial cells as a potential target of metronomic chemotherapy. Arch Pharm Res..

[10] Wildiers H (2007). Mastering chemotherapy dose reduction in elderly cancer patients. Eur J Cancer..

[11] Bocci G, Nicolaou KC, Kerbel RS (2002). Protracted low-dose effects on human endothelial cell proliferation and survival in vitro reveal a selective antiangiogenic window for various chemotherapeutic drugs. Cancer Res..

[12] Bocci G, Francia G, Man S, Lawler J, Kerbel RS (2003). Thrombospondin 1, a mediator of the antiangiogenic effects of low-dose metronomic chemotherapy. Proc Natl Acad Sci U S A..

[13] Browder T, Butterfield CE, Kräling BM, Shi B, Marshall B, O’Reilly MS (2000). Antiangiogenic scheduling of chemotherapy improves efficacy against experimental drug-resistant cancer. Cancer Res..

[14] Panthi VK, Dua K, Singh SK, Gupta G, Hansbro PM, Paudel KR (2023). Nanoformulations-based metronomic chemotherapy: mechanism, challenges, recent advances, and future perspectives. Pharmaceutics..

[15] Natale G, Bocci G (2018). Does metronomic chemotherapy induce tumor angiogenic dormancy? A review of available preclinical and clinical data. Cancer Lett..

[16] Orecchioni S, Talarico G, Labanca V, Calleri A, Mancuso P, Bertolini F (2018). Vinorelbine, cyclophosphamide and 5-FU effects on the circulating and intratumoural landscape of immune cells improve anti-PD-L1 efficacy in preclinical models of breast cancer and lymphoma. Br J Cancer..

[17] Maiti R (2014). Metronomic chemotherapy. J Pharmacol Pharmacother.

[18] Muthusamy P, Chary KV, Nalini GK. Metronomic chemotherapy: Seems prowess to battle against cancer in current scenario. J Clin Diagn Res. 2016;10:FC09-FC13. 10.7860/JCDR/2016/23782.8802 PMC5198346 28050393

[19] Bocci G, Tuccori M, Emmenegger U, Liguori V, Falcone A, Kerbel RS (2005). Cyclophosphamide-methotrexate “metronomic” chemotherapy for the palliative treatment of metastatic breast cancer. A comparative pharmacoeconomic evaluation. Annal Oncol.

[20] Cazzaniga ME, Munzone E, Bocci G, Afonso N, Gomez P, Langkjer S (2019). Pan-European expert meeting on the use of metronomic chemotherapy in advanced breast cancer patients: The Penelope project. Adv Ther..

[21] Bocci G, Di Paolo A, Danesi R (2013). The pharmacological bases of the antiangiogenic activity of paclitaxel. Angiogenesis..

[22] Banchi M, Lanzolla T, Di Napoli A, Bandini A, Bocci G, Cox MC. Complete remission of a diffuse large B-cell lymphoma in a young patient, with severe tuberous sclerosis, treated with metronomic chemotherapy and ibrutinib: a case report. Chemotherapy. 2023:1–5. 10.1159/000533236 37549660

[23] Riesco-Martinez M, Parra K, Saluja R, Francia G, Emmenegger U (2017). Resistance to metronomic chemotherapy and ways to overcome it. Cancer Lett..

[24.•] Sureshkumar K, Srinivasan K, Lingeshwaran GP, Durairaj M, Thiruvengadam G, Mary Martin Daniel PJ (2023). Toxicity profile, adverse drug reactions and drug-drug interactions among geriatric cancer patients under metronomic chemotherapy in a South Indian tertiary care hospital. J Oncol Pharm Pract.

[25] Kashyap D, Pal D, Sharma R, Garg VK, Goel N, Koundal D (2022). Global increase in breast cancer incidence: risk factors and preventive measures. Biomed Res Int..

[26] DeSantis CE, Ma J, Gaudet MM, Newman LA, Miller KD, Goding Sauer A (2019). Breast cancer statistics, 2019. CA Cancer J Clin..

[27] Di Desidero T, Xu P, Man S, Bocci G, Kerbel RS (2015). Potent efficacy of metronomic topotecan and pazopanib combination therapy in preclinical models of primary or late stage metastatic triple-negative breast cancer. Oncotarget..

[28] De Iuliis F, Salerno G, Taglieri L, Lanza R, Scarpa S (2015). On and off metronomic oral vinorelbine in elderly women with advanced breast cancer. Tumori..

[29.•] Wildiers H, Tryfonidis K, Dal Lago L, Vuylsteke P, Curigliano G, Waters S (2018). Pertuzumab and trastuzumab with or without metronomic chemotherapy for older patients with HER2-positive metastatic breast cancer (EORTC 75111-10114): an open-label, randomised, phase 2 trial from the Elderly Task Force/Breast Cancer Group. Lancet Oncol..

[30] Krajnak S, Decker T, Schollenberger L, Rosé C, Ruckes C, Fehm T (2021). Phase II study of metronomic treatment with daily oral vinorelbine as first-line chemotherapy in patients with advanced/metastatic HR+/HER2− breast cancer resistant to endocrine therapy: VinoMetro—AGO-B-046. J Cancer Res Clin Oncol..

[31] Addeo R, Sgambato A, Cennamo G, Montella L, Faiola V, Abbruzzese A (2010). Low-dose metronomic oral administration of vinorelbine in the first-line treatment of elderly patients with metastatic breast cancer. Clin Breast Cancer..

[32] Cazzaniga ME, Torri V, Riva F, Porcu L, Cicchiello F, Capici S (2017). Efficacy and safety of vinorelbine-capecitabine oral metronomic combination in elderly metastatic breast cancer patients: VICTOR-1 study. Tumori..

[33] Bottini A, Generali D, Brizzi MP, Fox SB, Bersiga A, Bonardi S (2006). Randomized phase II trial of letrozole and letrozole plus low-dose metronomic oral cyclophosphamide as primary systemic treatment in elderly breast cancer patients. J Clin Oncol..

[34] Crivellari D, Gray KP, Dellapasqua S, Puglisi F, Ribi K, Price KN (2013). Adjuvant pegylated liposomal doxorubicin for older women with endocrine nonresponsive breast cancer who are NOT suitable for a “standard chemotherapy regimen” : The CASA randomized trial. Breast..

[35] Fontana A, Falcone A, Derosa L, Di Desidero T, Danesi R, Bocci G (2010). Metronomic chemotherapy for metastatic prostate cancer: A “young”concept for old patients?. Drugs Aging..

[36] Parshad S, Sidhu AK, Khan N, Naoum A, Emmenegger U (2022). Metronomic chemotherapy for advanced prostate cancer: A literature review. J Clin Med..

[37] Simsek C, Esin E, Yalcin S (2019). Metronomic chemotherapy: A systematic review of the literature and clinical experience. J Oncol..

[38] Petrylak DP, Tangen CM, Hussain MHA, Lara PN, Jones JA, Taplin ME (2004). Docetaxel and estramustine compared with mitoxantrone and prednisone for advanced refractory prostate cancer. New Engl J Med.

[39] Tannock IF, de Wit R, Berry WR, Horti J, Pluzanska A, Chi KN (2004). Docetaxel plus prednisone or mitoxantrone plus prednisone for advanced prostate cancer. New Engl J Med.

[40.•] Fontana A, Bocci G, Galli L, D’Arcangelo M, Derosa L, Fioravanti A, et al. Metronomic cyclophosphamide in elderly patients with advanced, castration-resistant prostate cancer. J Am Geriatr Soc. 2010;58:986–8. **This retrospective study emphasized the efficacy and favorable toxicity profile of MCT in the treatment of mCRPC in elderly patients.** 10.1111/j.1532-5415.2010.02833.x 20722827

[41] Man S, Bocci G, Francia G, Green SK, Jothy S, Hanahan D (2002). Antitumor effects in mice of low-dose (metronomic) cyclophosphamide administered continuously through the drinking water. Cancer Res..

[42] Fontana A, Galli L, Fioravanti A, Orlandi P, Galli C, Landi L (2009). Clinical and pharmacodynamic evaluation of metronomic cyclophosphamide, celecoxib, and dexamethasone in advanced hormone-refractory prostate cancer. Clin Cancer Res.

[43] Tralongo P, Bordonaro S, Di Mari A, Cappuccio F, Rametta GS (2016). Chemotherapy in frail elderly patients with hormone-refractory prostate cancer: A “real world” experience. Prostate Int..

[44] Tagliamento M, Frelaut M, Baldini C, Naigeon M, Nencioni A, Chaput N (2022). The use of immunotherapy in older patients with advanced non-small cell lung cancer. Cancer Treat Rev..

[45] Owonikoko TK, Ragin CC, Belani CP, Oton AB, Gooding WE, Taioli E (2007). Lung cancer in elderly patients: An analysis of the surveillance, epidemiology, and end results database. J Clin Oncol.

[46] Planchard D, Popat S, Kerr K, Novello S, Smit EF, Faivre-Finn C, et al. Metastatic non-small cell lung cancer: ESMO clinical practice guidelines for diagnosis, treatment and follow-up. Ann Oncol 2018;29:iv192–237. 10.1093/annonc/mdy275 30285222

[47] Camerini A, Banna GL, Cinieri S, Pezzuto A, Mencoboni M, Rosetti F (2019). Metronomic oral vinorelbine for the treatment of advanced non-small cell lung cancer: a multicenter international retrospective analysis. Clin Transl Oncol.

[48] Estevinho F, Gomes R, Hasmucrai D, Barata F (2022). Metronomic oral vinorelbine in a real-world population of advanced non-small cell lung cancer patients. Pulmonology..

[49] Orlandi P, Di Desidero T, Salvia G, Muscatello B, Francia G, Bocci G (2018). Metronomic vinorelbine is directly active on non small cell lung cancer cells and sensitizes the EGFRL858R/T790M cells to reversible EGFR tyrosine kinase inhibitors. Biochem Pharmacol..

[50] Briasoulis E, Aravantinos G, Kouvatseas G, Pappas P, Biziota E, Sainis I (2013). Dose selection trial of metronomic oral vinorelbine monotherapy in patients with metastatic cancer: A hellenic cooperative oncology group clinical translational study. BMC Cancer..

[51] Gironés Sarrió R, Antonio Rebollo M, Molina Garrido MJ, Guillén-Ponce C, Blanco R, Gonzalez Flores E (2018). General recommendations paper on the management of older patients with cancer: the SEOM geriatric oncology task force’s position statement. Clin Transl Oncol.

[52] Pasini F, Barile C, Caruso D, Modena Y, Fraccon AP, Bertolaso L (2018). Oral Metronomic Vinorelbine (OMV) in elderly or pretreated patients with advanced non small cell lung cancer: outcome and pharmacokinetics in the real world. Invest New Drugs..

[53.•] D’D’Ascanio M, Pezzuto A, Fiorentino C, Sposato B, Bruno P, Grieco A, et al. metronomic chemotherapy with vinorelbine produces clinical benefit and low toxicity in frail elderly patients affected by advanced non-small cell lung cancer. Biomed Res Int. 2018;2018:6278403. **This study confirmed the efficacy in controlling cancer progression and good safety profile of metronomic VNR administered at different doses in elderly advanced NSCLC patients.** 10.1155/2018/6278403 PMC6129793 30225260

[54] Di M, Huntington SF, Olszewski AJ (2021). Challenges and opportunities in the management of diffuse large B-cell lymphoma in older patients. Oncologist..

[55.•] Cox MC, Banchi M, Pelliccia S, Di Napoli A, Marcheselli L, Patti C, et al. All-oral metronomic DEVEC schedule in elderly patients with peripheral T cell lymphoma. Cancer Chemother Pharmacol. 2020;86:841–6. **In this multicenter study the DEVEC schedule showed encouraging activity and acceptable toxicity for the treatment of elderly PTCL patients.** 10.1007/s00280-020-04172-3 PMC7568761 33070248

[56] Cox MC, Bocci G (2022). Metronomic chemotherapy regimens and targeted therapies in non-Hodgkin lymphoma: The best of two worlds. Cancer Lett..

[57.••] Cox MC, Musuraca G, Battistini R, Casaroli I, Zoli V, Anticoli-Borza P (2018). Aggressive lymphomas of the elderly: the DEVEC metronomic chemotherapy schedule fits the unfit. Br J Haematol.

[58] Rozados VR, Sánchez AM, Gervasoni SI, Berra HH, Matar P, Scharovsky OG (2004). Metronomic therapy with cyclophosphamide induces rat lymphoma and sarcoma regression, and is devoid of toxicity. Ann Oncol.

[59] Witte HM, Riecke A, Mayer T, Bartscht T, Rades D, Lehnert H (2019). Trofosfamide in the treatment of elderly or frail patients with diffuse large B-cell lymphoma. J Cancer Res Clin Oncol..

[60] Schelker RC, Herr W, Reichle A, Vogelhuber M (2018). Low-dose trofosfamide plus rituximab is an effective and safe treatment for diffuse large B-cell lymphoma of the elderly: A single center experience. BMC Cancer..

[61] Zeng J, Yang L, Huang F, Hong T, He Z, Lei J (2016). The metronomic therapy with prednisone, etoposide, and cyclophosphamide reduces the serum levels of VEGF and circulating endothelial cells and improves response rates and progression-free survival in patients with relapsed or refractory non-Hodgkin’s lymp. Cancer Chemother Pharmacol..

[62] Coleman M, Martin P, Ruan J, Furman R, Niesvizky R, Elstrom R (2008). Low-dose metronomic, multidrug therapy with the PEP-C oral combination chemotherapy regimen for mantle cell lymphoma. Leuk Lymphoma..

[63] Peyrade F, Jardin F, Thieblemont C, Thyss A, Emile JF, Castaigne S (2011). Attenuated immunochemotherapy regimen (R-miniCHOP) in elderly patients older than 80 years with diffuse large B-cell lymphoma: A multicentre, single-arm, phase 2 trial. Lancet Oncol..

[64] Peyrade F, Bologna S, Delwail V, Emile JF, Pascal L, Fermé C (2017). Combination of ofatumumab and reduced-dose CHOP for diffuse large B-cell lymphomas in patients aged 80 years or older: an open-label, multicentre, single-arm, phase 2 trial from the LYSA group. Lancet Haematol..

[65] Cox MC, Pelliccia S, Marcheselli L, Battistini R, Arcari A, Borza PA (2019). The metronomic all-oral DEVEC is an effective schedule in elderly patients with diffuse large b-cell lymphoma. Invest New Drugs..

[66.••] Bocci G, Pelliccia S, Orlandi P, Caridi M, Banchi M, Musuraca G, et al. Remarkable remission rate and long-term efficacy of upfront metronomic chemotherapy in elderly and frail patients, with diffuse large B-cell lymphoma. J Clin Med. 2022:11. **This multicenter study confirmed that the oral metronomic protocol DEVEC exerts an effective pharmacological activity on cancer cells that allows to obtain a lasting remission and long-term effectiveness, with acceptable toxicity and good tolerability in elderly and fragile patients with DLBCL.** 10.3390/jcm11237162 PMC9739472 36498736

[67] Fante MA, Harrer DC, Zartner B, Lüke F, Mayer S, Menhart K (2023). All-oral low-dose chemotherapy TEPIP is effective and well-tolerated in patients with peripheral T-cell lymphoma. Front Oncol..

[68] Ruan J, Martin P, Coleman M, Furman RR, Cheung K, Faye A (2010). Durable responses with the metronomic rituximab and thalidomide plus prednisone, etoposide, procarbazine, and cyclophosphamide regimen in elderly patients with recurrent mantle cell lymphoma. Cancer..

[69] Borne E, Desmedt E, Duhamel A, Mirabel X, Dziwniel V, Maire C (2010). Oral metronomic cyclophosphamide in elderly with metastatic melanoma. Invest New Drugs..

[70] Dinkic C, Eichbaum M, Schmidt M, Grischke EM, Gebauer G, Fricke HC (2017). Pazopanib (GW786034) and cyclophosphamide in patients with platinum-resistant, recurrent, pre-treated ovarian cancer - Results of the PACOVAR-trial. Gynecol Oncol..

[71] He S, Shen J, Hong L, Niu L, Niu D (2012). Capecitabine “metronomic” chemotherapy for palliative treatment of elderly patients with advanced gastric cancer after fluoropyrimidine-based chemotherapy. Med Oncol..

[72] Liu J, Wang Y, Jiang H, Yu X, Xu N (2019). Long-term survival of an elderly female with metastatic colon cancer after treated with capecitabine monotherapy. Medicine..

[73] Muratori L, La SA, Gorzegno G, Sperone P, Scagliotti GV (2020). Long-term disease control in a metastatic squamous cell carcinoma of the oral cavity treated with maintenance metronomic capecitabine. J Oncol Pharm Pract.

[74] Phinyo P, Patumanond J, Pongudom S (2021). Time-dependent treatment effects of metronomic chemotherapy in unfit AML patients: a secondary analysis of a randomised controlled trial. BMC Res Notes..

[75] Hartmann JT, Kopp HG, Gruenwald V, Piperno-Neumann S, Kunitz A, Hofheinz R (2020). Randomised phase II trial of trofosfamide vs. doxorubicin in elderly patients with untreated metastatic soft-tissue sarcoma. Eur J Cancer..

